# Monocarbonyl Curcumin Analogues as Potent Inhibitors against Human Glutathione Transferase P1-1

**DOI:** 10.3390/antiox12010063

**Published:** 2022-12-28

**Authors:** Panagiota Pantiora, Veronika Furlan, Dimitris Matiadis, Barbara Mavroidi, Fereniki Perperopoulou, Anastassios C. Papageorgiou, Marina Sagnou, Urban Bren, Maria Pelecanou, Nikolaos E. Labrou

**Affiliations:** 1 Laboratory of Enzyme Technology, Department of Biotechnology, School of Applied Biology and Biotechnology, Agricultural University of Athens, 75 Iera Odos Street, GR-11855 Athens, Greece; 2 Institute of Biosciences & Applications, NCSR “Demokritos”, 15310 Athens, Greece; 3 Faculty of Chemistry and Chemical Engineering, University of Maribor, Smetanova 17, SI-2000 Maribor, Slovenia; 4 Turku Bioscience Centre, University of Turku and Åbo Akademi University, 20521 Turku, Finland; 5 Faculty of Mathematics, Natural Sciences and Information Technologies, University of Primorska, Glagoljaška 8, SI-6000 Koper, Slovenia; 6 Institute of Environmental Protection and Sensors, Beloruska Ulica 7, SI-2000 Maribor, Slovenia

**Keywords:** curcuminoids, curcumin analogues, human glutathione transferase P1-1 (hGSTP1-1), glutathione transferase, enzyme inhibition, multi-drug resistance

## Abstract

The isoenzyme of human glutathione transferase P1-1 (hGSTP1-1) is involved in multi-drug resistance (MDR) mechanisms in numerous cancer cell lines. In the present study, the inhibition potency of two curcuminoids and eleven monocarbonyl curcumin analogues against hGSTP1-1 was investigated. Demethoxycurcumin (Curcumin II) and three of the monocarbonyl curcumin analogues exhibited the highest inhibitory activity towards hGSTP1-1 with IC_50_ values ranging between 5.45 ± 1.08 and 37.72 ± 1.02 μM. Kinetic inhibition studies of the most potent inhibitors demonstrated that they function as non-competitive/mixed-type inhibitors. These compounds were also evaluated for their toxicity against the prostate cancer cells DU-145. Interestingly, the strongest hGSTP1-1 inhibitor, (DM96), exhibited the highest cytotoxicity with an IC_50_ of 8.60 ± 1.07 μΜ, while the IC_50_ values of the rest of the compounds ranged between 44.59–48.52 μΜ. Structural analysis employing molecular docking, molecular dynamics (MD) simulations, and binding-free-energy calculations was performed to study the four most potent curcumin analogues as hGSTP1-1 inhibitors. According to the obtained computational results, DM96 exhibited the lowest binding free energy, which is in agreement with the experimental data. All studied curcumin analogues were found to form hydrophobic interactions with the residue Gln52, as well as hydrogen bonds with the nearby residues Gln65 and Asn67. Additional hydrophobic interactions with the residues Phe9 and Val36 as well as π–π stacking interaction with Phe9 contributed to the superior inhibitory activity of DM96. The van der Waals component through shape complementarity was found to play the most important role in DM96-inhibitory activity. Overall, our results revealed that the monocarbonyl curcumin derivative DM96 acts as a strong hGSTP1-1 inhibitor, exerts high prostate cancer cell cytotoxicity, and may, therefore, be exploited for the suppression and chemosensitization of cancer cells. This study provides new insights into the development of safe and effective GST-targeted cancer chemosensitizers.

## 1. Introduction

Glutathione transferases (GSTs) constitute a multigene family of detoxifying enzymes, which participate in phase II metabolism [1,2,3,4]. These enzymes mainly catalyze the conjugation of various toxic electrophilic or xenobiotic compounds with the reduced form of glutathione tripeptide (GSH) [3,5,6]. Several crystallographic studies have demonstrated that GSTs are dimeric proteins. In each subunit, two distinct binding sites are located: one for the glutathione (G-site) and the other for the electrophilic substrate (H-site) [7,8]. Human cytosolic GSTs are classified into seven classes, namely alpha (A), zeta (Z), theta (T), mu (M), pi (P), sigma (S), and omega (O) [9,10,11]. Recently, GSTs have attracted attention in the medical field as several GST isoenzymes, especially hGSTP1-1, are overexpressed in various types of cancer [12,13,14,15], resulting in the development of resistance to chemotherapeutic drugs and subsequent treatment failure [5,9,16]. hGSTP1-1 is the most widely studied member of the GST family. It is involved in apoptosis resistance and the metabolism of several chemotherapeutic agents, such as platinum-based drugs [17]. Furthermore, hGSTP1-1 modulates the function of apoptotic signaling of Jun-kinase [18] and Bax [19] and regulates calcium channels by decreasing the apoptotic mobilization of calcium ions [20]. In addition, hGSTP1-1 plays an important role in the regulation of tumor necrosis factor-α (TNF-α), TNF-receptor factor 2 (TRAF2), and apoptosis signal-regulating kinase 1 [21]. Both activator protein 1 and nuclear factor (NF)-κB mediate the regulation of hGSTP1-1 through a redox process [22].

In order to reduce or even eliminate this undesired activity of GSTs, a variety of synthetic or natural compounds have been evaluated for their inhibitory potentials against a range of GST isoenzymes. Recent examples include: 2,2′-dihydroxybenzophenones [9,13,23], benzoxazole [24], selenium compounds [25], diselenides and benzisoselenazolones [26], benzoxadiazoles [27,28], auranofin [29], ethacrynic acid [30], piperlongumine [31], and curcumin and curcumin analogues [32].

Curcumin is the major curcuminoid extracted from the rhizome of the *Curcuma longa* plant [33]. The other two curcuminoids, namely demethoxycurcumin and bisdemethoxycurcumin, are much less abundant and less investigated [33,34]. Curcumin is a widely studied compound with a broad spectrum of pharmacological and biological properties, including anticancer activity against numerous types of cancer [35,36]. Larasati et al. [32] have reported that curcumin inhibits tumor growth by increasing ROS levels over the threshold through which ROS-metabolizing enzymes (e.g., carbonyl reductase, glutathione transferase, glyoxalase, etc.) can mitigate. Another ROS-related therapeutic strategy involving curcumin and its derivatives is the anticancer photodynamic therapy application, in which increased ROS production is achieved as a result of curcumin photoactivation [37]. Therefore, curcumin has a potential in therapy to regulate ROS levels in tumor cells, thereby controlling tumor growth [38]. However, its limited bioavailability, aqueous solubility, and stability have prompted scientists to develop curcumin analogues with a more favorable pharmacological profile [39]. One of the strategies to achieve this goal is through the development of monocarbonyl curcumin analogues [40]. Several monocarbonyl analogues of curcumin (MACs) have shown improved chemical and metabolic stability related to bioavailability [34,41], as well as increased intestinal permeability and water solubility [42,43]. Although several studies have demonstrated the ability of curcumin to reduce the expression of GST genes and inhibit the activity of the main cytosolic isoenzymes hGSTA1, hGSTA2, hGSTM1, hGSTM2, and hGSTP1 [44,45,46], only a few monocarbonyl curcumin analogues have been evaluated for their inhibitory activity against the GST cytosolic isoenzymes [47,48]. For instance, Appiah-Opong et al. [48] investigated the inhibitory effect of 34 monocarbonyl curcumin analogues, categorized as substituted 2,6-dibenzylidenecyclohexanones, 2,5-dibenzylidenecyclopentanones, and 1,4-pentadiene-3-ones, on three human GST isoenzymes, namely hGSTA1-1, hGSTM1-1, and hGSTP1-1. Most of the 34 curcumin analogues showed lower inhibitory activity against hGSTA1-1, hGSTM1-1, and hGSTP1-1 than the parent curcumin. hGSTP1-1 was predominantly strongly inhibited by some 1,4-pentadiene-3-one compounds with IC_50_ values between 0.4–4.6 μM [48]. In other works, van Iersel et al. [49] and Hayeshi et al. [44] have reported that, at pH 7.4, curcumin behaves as an irreversible inhibitor against hGSTP1-1, presumably by covalent binding to Cys-47.

**Figure 1:**
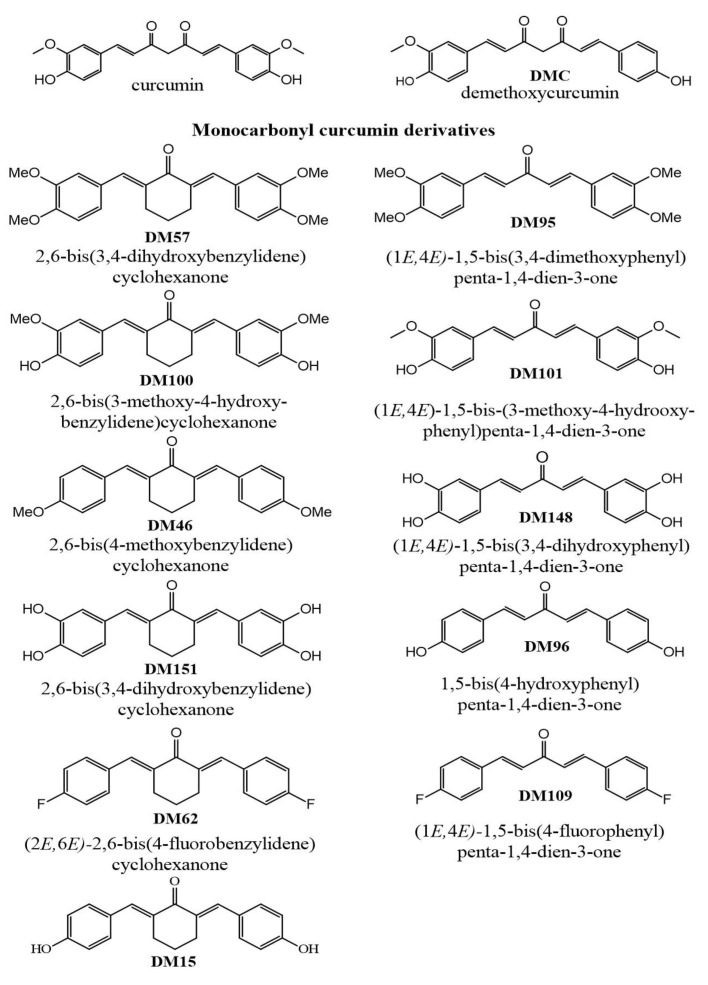
Structures, names, and codes of curcuminoids and of the monocarbonyl curcumin derivatives used in the present study.

Herein, the inhibition potency of two curcuminoids, curcumin and demethoxycurcumin, and eleven monocarbonyl curcumin analogues against hGSTP1-1 was evaluated. Furthermore, their cytotoxicity against the prostate cancer cell line DU-145, which overexpresses this isoenzyme [50], was also investigated. To the best of our knowledge there is no previous report on the hGSTP1-1 inhibitory activity of demethoxycurcumin. The rest of the tested compounds are mostly mono- and disubstituted monocarbonyl analogues with various hydroxy- and methoxy- group substitution patterns and one fluoro-substitution (Figure 1). Some of the tested compounds additionally allowed for the investigation of the effect of a central cyclohexanone core, compared to the acetone skeleton, on the hGSTP1-1 inhibitory activity.

## 2. Materials and Methods

### 2.1. Materials

#### 2.1.1. Chemicals

Reduced GSH, 1-chloro-2,4-dinitrobenzene (CDNB), ampicillin, sodium dodecyl sulfate (SDS), and the chromatographic material Sepharose CL-6B were purchased from Sigma-Aldrich, St. Louis, MO, USA (Merck, Rahway, NJ, USA) and were used without further treatment. Ethanol, methanol, and dimethyl sulfoxide (DMSO) were purchased from Scharlau (Barcelona, Spain). DU-145 culture media were obtained from Thermo Fisher Scientific (Waltham, MA, USA). The (3-[4,5-dimethylthiazol-2-yl]-2,5-diphenyl-tetrazolium bromide) reagent (MTT) was purchased from Applichem (Darmstadt, Germany).

#### 2.1.2. Bacterial Strains, Plasmids, and Cancer Cell Line

*Escherichia coli* (*E. coli*) strain BL21 (DE3) was utilized as an expression host for the production of the recombinant enzyme hGSTP1-1. The plasmid pEXP5-CT/TOPO^®^ was supplied by Invitrogen, Carlsbad, CA, USA. DU-145 human prostate cancer cell line was obtained from the cell bank of the Institute of Biosciences & Applications, NCSR “Demokritos”, and was free of mycoplasma contamination as judged visually under microscope observation and by 4′,6-diamidino-2-phenylindole (DAPI) staining of the cell cultures. The media/agents for the cell cultures were purchased from Thermo Fisher Scientific (Waltham, MA, USA). For the MTT experiments, absorbance was recorded by Sirio S Seac RADIM-Group Diachel ELISA plate reader.

#### 2.1.3. Curcuminoids and Curcumin Analogues

The curcuminoids and curcumin analogues evaluated as potential inhibitors of hGSTP1-1 isoenzyme were synthesized according to reported procedures [51,52,53,54]. For the synthesis of DM109 ((1E,4E)-1,5-bis(4-fluorophenyl)penta-1,4-dien-3-one), a modified synthetic procedure was employed, resulting in better yield and higher purity [51], as follows: 4-fluorobenzaldehyde (0.62 g, 5.0 mmol) was dissolved in absolute EtOH (5 mL). Acetone (0.19 mL, 2.5 mmol) was added to this solution and then a solution of sodium ethoxide (0.17 g, 2.5 mmol) in EtOH (1 mL) was added dropwise. The resulting mixture was stirred at room temperature for 2 h. The resultant precipitate was filtered under vacuum, washed with water and cold EtOH/water mixture (7:3), and dried under vacuum and P_2_O_5_. The pure product was obtained as yellow powder. Yield: 0.45 g (67%), 1H NMR (500 MHz, DMSO-d6) δ: 7.29–7.33 (m, 6H), 7.80 (d, J = 16.0 Hz, 2H), 7.86 (d, J = 8.6 Hz, 2H), 7.88 (d, J = 8.6 Hz, 2H). Further information on the synthesis and NMR characteristics of the compounds are provided in the Supplementary Material.

### 2.2. Methods

#### 2.2.1. Expression and Purification of hGSTP1-1 from Recombinant *E. coli* Cells

The expression and purification of the hGSTP1-1 were based on a published method [9,22]. The purity of the enzyme was evaluated by sodium dodecyl sulphate–polyacrylamide gel electrophoresis (SDS-PAGE). Purified enzyme fractions were pooled, diluted by dropwise addition of glycerol (to 50% *v*/*v* final concentration), and stored at −20 °C.

#### 2.2.2. Protein Determination

Protein concentration was determined according to Bradford assay using bovine serum albumin as a standard [55].

#### 2.2.3. Enzyme Assays and Inhibition Studies

Determination of hGSTP1-1 activity was performed by monitoring the formation of the conjugate between CDNB and GSH and measuring the absorption at λ = 340 nm (ε = 9600 L·mol^−1^·cm^−1^) at 25 °C for 120 s, as described previously [9]. One unit of enzyme activity is defined as the amount of enzyme that produces 1.0 μmol of product per minute under the assay conditions. Curcuminoids and monocarbonyl curcumin analogues were dissolved in DMSO (100 μΜ) and added to the assay mixture (the volume of DMSO was maintained at 2% *v*/*v* final concentration). The mixture was incubated at 25 °C for 1 min, prior to adding the enzyme sample. Initial velocities were determined in triplicates and were corrected for spontaneous reaction rates, when necessary. For the determination of IC_50_ values for the most potent inhibitors (DMC, DM96, DM109, and DM151), the assay mixture was identical to the one described above, in the presence of different concentrations of inhibitors. The IC_50_ values were determined from a graph of the remaining GST activity (%) against inhibitor concentration.

The computer program GraphPad Prism version 8 (GraphPad Prism Software, Inc., San Diego, CA, USA) was used for producing kinetic graphs and determining apparent kinetic parameters/constants and IC_50_ values.

#### 2.2.4. Kinetic Inhibition Analysis

Initial velocities (25 °C) for the hGSTP1-1-catalyzed reaction with CDNB as a variable substrate were determined in reaction mixtures (1 mL total volume) containing potassium phosphate buffer (100 mM, pH 6.5), 2.5 mM GSH, and different concentrations of CDNB (typically 14–1000 μM) in the absence and presence of inhibitors (DM96, DM151, DM109, DMC, 0–50 μM). Initial velocities for the hGSTP1-1-catalyzed reaction with GSH as a variable substrate were determined in reaction mixtures (1 mL total volume) (25 °C) containing potassium phosphate buffer (100 mM, pH 6.5), 1 mM CDNB, and different concentrations of GSH (typically 37.5–3750 μΜ) in the absence and presence of inhibitors (DM96, DM151, DM109, Curcumin II, 0–50 μM).

#### 2.2.5. Cell Cultures

DU-145 cells were grown in Dulbecco’s modified eagle medium (DMEM) growth medium of pH 7.4, supplemented with 10% fetal bovine serum (FBS), penicillin (100 U·mL^−1^), glutamine (2 mM), and streptomycin (100 μg·mL^−1^). Cell cultures were maintained in flasks and were grown at 37 °C in a humidified atmosphere of 5% CO2 in air. Sub-confluent cells were detached using a 0.05% (*w*/*v*) trypsin–0.25% (*w*/*v*) ethylenediaminetetraacetic acid (EDTA) solution. The subcultivation ratio was 1:2 to 1:5.

##### In Vitro Cytotoxicity Evaluation of Curcumin Analogues against DU-145 Cells

In vitro cytotoxicity of DMC, DM96, DM109, and DM151 against DU-145 cell lines was determined by the MTT colorimetric assay as previously described [56]. Cells were seeded in 96-well plates (8 × 10^3^ cells/well in 100 μL culture medium) and grown overnight at 37 °C in a 5% CO2 incubator. Exponentially growing cells were incubated for 48 h with various concentrations ranging between 10^−3^–10^−8^ μM of each derivative, and the final DMSO concentration never exceeded 0.2%. The medium was then removed and replaced with 100 μL of MTT solution (1 mg·mL^−1^). After a 4 h incubation, the solution was aspirated, formazan crystals were solubilized in 100 μL of DMSO, and absorbance was recorded at 540 nm on a Tecan well plate reader. The results were expressed as % cell viability = (mean optical density (OD) of treated cells/mean OD of untreated cells) × 100. Data were plotted against the corresponding compound concentration in a semi-log chart and the values of IC_50_ (the concentration of test compound required to reduce the fraction of live cells to 50% of the control) were calculated from the dose–response curves using GraphPad Prism 8 software.

##### Inhibition Studies of Curcumin Analogues against Native GST Extracted from DU-145 Cells

DU-145 cells were trypsinized, transferred to a Falcon tube, and centrifuged at 1500× *g* for 10 min. Subsequently, DMEM was removed and the cell pellet was resuspended in 1 mL of potassium phosphate buffer. The cells were lysed by sonication, the suspension was centrifuged at 13,000× *g* for 5 min, and the supernatant was collected. Inhibition of native DU-145-extracted GST activity by DMC, DM96, DM109, and DM151 was measured at 25 °C as described above for the recombinant enzyme. The concentration of inhibitors was adjusted to equal their IC_50_ values determined using the recombinant hGSTP1-1.

#### 2.2.6. Circular Dichroism (CD) Studies

CD studies were performed using a JASCO J-715 spectropolarimeter (Jasco Co., Tokyo, Japan) equipped with Peltier temperature controller (Jasco Co., Tokyo, Japan), at 25 °C and a wavelength range of 190–400 nm using a 1-mm-path length quartz cuvette. Each CD spectrum is the average of three scans at 200 nm·min^−1^ and a resolution of 0.5 nm. The CD solutions were prepared by mixing the appropriate volumes of stock solutions to give final concentrations of 0.1 mg/mL of hGSTP1-1, 2.5 mM of GSH, 1 mM of CDNB, and 5 μΜ of inhibitor in potassium phosphate buffer (100 mM, pH 6.5) at room temperature, while CD spectra of plain solutions of GSH, CDNB, DM96, and DM62 at the same concentrations were also run as controls (see supporting information). The analysis of the CD data was performed using the OriginPro 9 program and the content of the secondary structure was calculated through the CDNN Circular Dichroism Spectroscopy Deconvolution software.

#### 2.2.7. Molecular Docking, Molecular Dynamics Simulations, and Free-Energy Calculations

##### Molecular Docking

To generate the starting models of hGSTP1-1 in complex with the four studied curcumin analogues, the docking protocol based on the CANDOCK algorithm was carried out [57]. The obtained poses were evaluated by the radial-mean-reduced scoring function at a cutoff radius of 6 Å from each atom of the ligand (RMR6). The co-ordinates of the X-ray crystal structure of hGSTP1-1 were obtained from the protein data bank (PDB ID: 18GS, chain A). The 3D structures of investigated curcumin analogues were prepared with Avogadro [58] and subsequently optimized with Gaussian 16 [59] in conjunction with the Hartree–Fock method and 6-31G(d) basis set. As the experimental crystal structures of DM96, DM151, DM109, and DMC in complex with hGSTP1-1 have not been determined yet, the binding modes of the studied analogues with the lowest docking score values were used for subsequent molecular dynamics (MD) simulations in conjunction with free-energy calculations.

##### Preparation of Initial Complexes for Molecular Dynamics Simulations

Preparation of the initial structure as well as molecular dynamics simulations and free-energy calculations were performed with the Q5 program package [60] using the AMBER [61] and GAFF force fields [62]. After molecular docking, the studied curcumin analogues were extracted from the complex with hGSTP1-1, and hydrogen atoms were added using Pymol [63]. The studied curcumin analogues were then subjected to full geometry optimization and subsequent vibrational analysis in the harmonic approximation at the Hartree–Fock (HF) level of theory in conjunction with 6-31G(d) basis set encoded in Gaussian 16 program [59]. The absence of imaginary vibrational frequencies indicated correctly performed minimizations. The restricted electrostatic charge fitting procedure (RESP) was initiated to reproduce the HF/6-31G(d)-calculated electrostatic potential (ESP) surrounding the studied compounds [64]. Partial charges of chemically equivalent atoms were restricted to the same value. The AMBER ff14 force field [61] was used for the parametrization of hGSTP1-1, while the general AMBER force field (GAFF) [62] was used for parametrization of the studied curcumin analogues in Antechamber.

The systems representing the ligand’s bound states were prepared by constructing a sphere of TIP3P [61] water molecules with a radius of 25 Å, centered in the center of mass of the studied ligands. The configurations of ligand’s free states were prepared by constructing a sphere of TIP3P water molecules around the ligand, again with a radius of 25 Å, and centered in the center of mass of the ligands. In the area between 22 Å and 25 Å away from the sphere center as well as outside the simulation sphere, the ionizable residues were generally treated as neutral entities [65,66]. The ionizable residues located within 22 Å of the sphere center were assigned their protonation states at pH 7.4 with the H++ software [67]. The sum of total charges in the bound state required the substitution of 3 water molecules with 3 chloride ions to achieve electroneutrality of the studied complexes. Topology and co-ordinate files necessary for the initiation of molecular dynamics simulations were generated using the Qprep5 program.

##### Molecular Dynamics Simulations

Subsequent molecular dynamics simulations and free-energy calculations were performed with the Q program package [60] using AMBER ff14 [68] and GAFF [62] force fields as well as the Linear Interaction Energy (LIE) and Linear Response Approximation (LRA) methods. Firstly, all solvated complexes were equilibrated by employing a series of 12 MD simulations in four parallels using different random seeds with the Qdyn5 program. The equilibrating MD simulation protocol was identical to the protocol in our previous study [69,70]. The values of stepsize, temperature, and external bath coupling parameters were gradually increased from 0.01 to 2 fs, 5 to 298.15 K, and 0.1 to 40 fs, respectively. The equilibrated complexes were then subjected to MD simulations. After equilibration of 183 ps length, 32 production runs of 20 ns each in an (N, V, T) ensemble at a temperature of 298.15 K were initiated from four independent starting configurations based on different random seeds. The integration step was 2 fs and the SHAKE algorithm was applied to bonds involving hydrogen atoms. The energy of the system was sampled at every 10th step and co-ordinate trajectories every 500th step. Non-bonding interactions were explicitly evaluated for distances shorter than 10 Å. Protein atoms protruding beyond the 25 Å sphere boundary were restrained to their co-ordinates from the crystal structure with non-bonding interactions turned off as well. Long-range electrostatics for distances beyond the 10 Å cut-off were treated with the local reaction field (LRF) method [71]. TIP3P molecules were subjected to the surface constraint all-atom solvent (SCAAS)-type boundary conditions designed to mimic an infinite aqueous solution [72]. Similarly, MD simulations in the free state were carried out using curcumin analogues in the water sphere in the absence of the protein. Visualization of co-ordinate trajectories was performed using the VMD [73] and Pymol [63] programs. The analysis of production trajectories was performed with MD analysis [74].

##### Binding-Free-Energy Calculations Using Linear Interaction Energy (LIE) and Linear Response Approximation (LRA) Methods

Binding free energies between curcumin analogues and their surroundings were calculated with the Qfep5 program for the free and the bound states using Linear Interaction Energy (LIE) and Linear Response Approximation (LRA) methods. The Linear Interaction Energy (LIE) approach [75] is based on linear response for treating electrostatic interactions and on an empirical term treating the non-polar interactions. Binding free energy can be calculated using Equation (1):

(1) $$\Delta G_{binding}^{L-P}=\alpha \left(\langle V_{vdW}^{L-P}\rangle_{q}-\langle V_{vdW}^{L-W}\rangle_{q}\right)+\beta \left(\langle V_{ele}^{L-P}\rangle_{q}-\langle V_{ele}^{L-W}\rangle_{q}\right)$$

where $\langle V_{vdW}^{L-P}\rangle_{q}$, $\langle V_{vdW}^{L-W}\rangle_{q}$, $\langle V_{ele}^{L-P}\rangle_{q}$, and $\langle V_{ele}^{L-W}\rangle_{q}$ represent average van der Waals and electrostatic interaction energies between ligand L and solvated binding site P or aqueous solution W during MD simulations. The subscript q indicates regular partial atomic charges of the ligand. α and β represent coefficients of the LIE method. Åqvist et al. [76] have found that α of 0.5 and β of 0.16 produce binding free energies in good agreement with experimental values for several protein systems [77]. However, Paulsen and Ornstein [78] found that refined values for the scaling factors α and β of 0.5 and 1.043, respectively, resulted in reasonable estimates of the binding free energies of 11 substrates binding to cytochrome P450cam concerning the corresponding experimental results. Therefore, it is reasonable to assume that the value of β is binding-site-dependent as it represents the scaling factor of van der Waals interactions, while the common value of α equals 0.5 [79]. In the LRA methodology [69,80], the additionally included preorganized electrostatics term $\langle V_{ele}^{L-P}\rangle_{0}$ represents the average electrostatic interaction energy between the ligand L and the solvated protein binding site P calculated over an ensemble of configurations generated by MD simulation using a ligand L in which all partial atomic charges have been set to zero [80]. In proteins, the binding-site dipoles associated with polar groups and ionized residues may be already partially oriented toward the bound ligand L with all partial atomic charges set to zero. The instantaneous charging of the bound ligand may consequently yield favorable electrostatic interactions with the binding-site amino acid residues ($\langle V_{ele}^{L-P}\rangle_{0}$ < 0). Proteins are, therefore, electrostatically preorganized to accommodate bound ligands. The binding free energy of a ligand L bound on the solvated protein binding site P can, therefore, be expressed as a linear combination of interaction energies presented in Equation (2):

(2) $$\Delta G_{binding}^{L-P}=\alpha \left(\langle V_{vdW}^{L-P}\rangle_{q}-\langle V_{vdW}^{L-W}\rangle_{q}\right)+\beta \left(\langle V_{ele}^{L-P}\rangle_{q}-\langle V_{ele}^{L-W}\rangle_{q}\right)+\beta \langle V_{ele}^{L-P}\rangle_{0}$$

As in the LIE method, the value of α equals 0.5, while the value of β was set to 1.043 [78,79]. Therefore, to apply LIE and LRA methodology and calculate the corresponding binding free energies, MD simulations on two physical states—the bound and the free state of an investigated ligand—had to be performed for the four studied curcumin analogues.

## 3. Results and Discussion

### 3.1. Screening of the Curcuminoids and Curcumin Analogues and Selection of the Most Potent Inhibitors

Recombinant hGSTP1-1 was expressed in *E. coli* BL21 cells and was purified to apparent homogeneity with a 38% yield using affinity chromatography on the GSH–Sepharose column [3,9]. The inhibitory potency of the curcumin analogues (Figure 1) against hGSTP1-1 was assessed at a final concentration of 100 μM, using enzyme activity assays [9]. The results (Table 1) allowed the identification of three distinct groups of inhibitory activities. The lowest inhibition potency was observed by the compounds DM62, DM46, and DM57, with inhibition ranging between 8.40–16.75%. The compounds DM100, DM95, Curcumin, DM148, DM15, and DM101 exhibited medium inhibition potency (40.0–72.28%), whereas the compounds DM109, DM96, DM151, and DMC showed the highest potency, reaching almost 95% inhibition, and were selected for further study.

These results indicate that certain structural features may be related to the hGSTP1-1 inhibition potential exhibited by the tested compounds. In particular, in the case of the monocarbonyl analogues, the presence of a methoxy-bearing aromatic ring results in the reduction of the inhibition potential, accentuating the importance of the presence of free hydroxyl group(s) for inhibition potency. Similarly, the presence of a cyclohexanone middle ring appears to negatively affect the inhibitory activity compared to the acetone analogues. This conclusion results from the observation that the inhibition caused by DM95 and DM101 is significant higher compared to that obtained by DM57 or DM100. The differentiation may be related to the increased hydrophobicity and volume occupancy of these molecules, obstructing their interaction with the enzyme. Similarly, the p-fluoro-containing cyclohexanone derivative DM62 exhibited very low inhibition activity whereas the acetone derivative DM109 was amongst the most potent compound tested, a difference possibly related to the more flexible structure of DM109 compared to the more rigid DM62.

**Figure 2:**
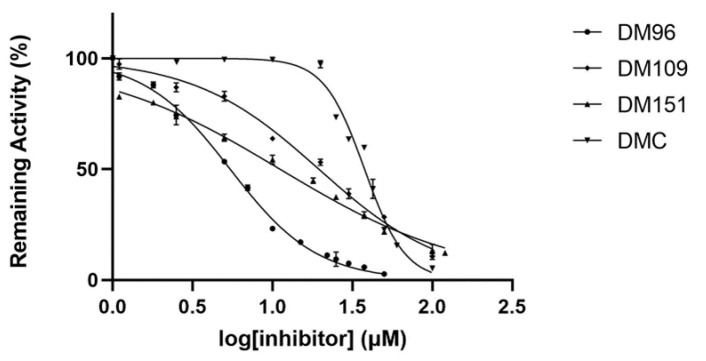
Concentration–response curves for the determination of the IC_50_ values of the most potent inhibitors DM96, DM109, DM151, and DMC against hGSTP1-1.

The compounds with the highest inhibition potency were thus selected for further dose–response studies for IC_50_ determinations. The results are depicted in Figure 2 and the corresponding IC_50_ values are listed in Table 2. The compound DM96 appears to be the strongest inhibitor against hGSTP1-1, with an IC_50_ value of 5.45 ± 1.08 μΜ. This was followed by DM151, DM109, and DMC with IC_50_ values of 11.17 ± 1.03 μΜ, 19.53 ± 1.04 μΜ, and 37.72 ± 1.02 μΜ, respectively.

### 3.2. Kinetic Inhibition Studies of hGSTP1-1 with the Curcumin Analogues DMC, DM96, DM109, and DM151

**Figure 3:**
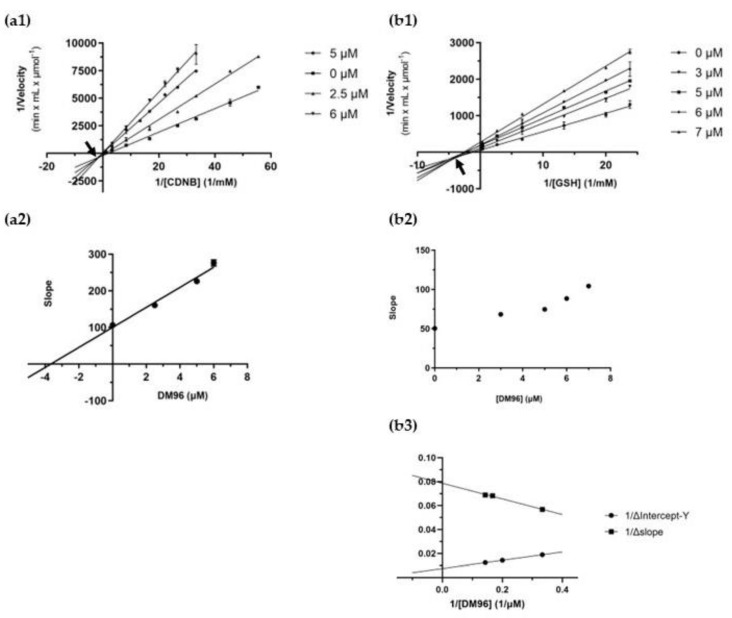
Kinetic inhibition studies. (**a1**) Lineweaver–Burk plot of the inhibition of hGSTP1-1 isoenzyme using CDNB as a variable substrate (18–980 μΜ) at different constant concentrations of DM96 (0, 2.5, 5, and 6 μM). (**a2**) Secondary plot of the slopes of each Lineweaver–Burk line as a function of DM96 concentration. (**b1**) Lineweaver–Burk plot of the inhibition of hGSTP1-1 isoenzyme using GSH as a variable substrate (37.5–3750 μΜ) at different constant concentrations of DM96 (0, 3, 5, 6, and 7 μM). (**b2**) Secondary plot of the slopes of each Lineweaver–Burk line as a function of DM96 concentration. (**b3**) Tertiary plot depicting the 1/ΔIntercept -Y and 1/Δslope as a function of the 1/[inhibitor].

The most potent inhibitors against hGSTP1-1 (DMC, DM96, DM109, and DM151) were further subjected to kinetic inhibition studies with the aim of determining the type of inhibition caused by the curcumin analogues and their mode of binding to the target enzyme. The results are listed in Table 2 and their graphical representation is shown in Figure 3. When CDNB was used as a variable substrate, DM96 appears to behave as a mixed-type inhibitor (Figure 3a1), and the slopes of each Lineweaver–Burk plot as a function of the inhibitor concentration follows a linear relationship (R^2^ = 0.98) (Figure 3a2). These results are consistent with the linear mixed-type inhibition model and suggest that DM96 can bind to both the free enzyme, with the inhibition constant K_i_ = 3.67 ± 0.35 μΜ, and to the enzyme/CDNB complex, with Κ_i’_ = 4.97 ± 2.86 μΜ. Using GSH as a variable substrate, DM96 again behaved as a mixed inhibitor, as evidenced by the Lineweaver–Burk graph (Figure 3b1). However, the slopes of each Lineweaver–Burk plot as a function of the inhibitor concentration follows a parabolic dependence, implying that the inhibition in this case is partially mixed (Figure 3b2). The observed non-linearity of the second plot allowed the generation of a linear third plot, that depicts the 1/ΔIntercept -Y and 1/Δslope as a function of the 1/[inhibitor] (Figure 3b3). These findings suggest that the inhibitor can bind to both the free enzyme and the enzyme–substrate complex, with K_i_ = 3.69 ± 1.00 μΜ and Κ_i’_ = 1.45 ± 0.43 μΜ, respectively. The partial mixed-type inhibition model observed indicates that DM96, upon inhibition, is either bound to the free enzyme or to the enzyme–substrate complex. Under these conditions, hGSTP1-1 is not fully inhibited and can produce product by either ES or ESI. The Lineweaver–Burk graphs of the other inhibitors DMC, DM151, and DM109 are illustrated in Figures S1 and S2, and the kinetic inhibition constants are listed in Table 2.

### 3.3. Cytotoxicity Studies of the Most Potent Inhibitors against DU-145 Prostate Cancer Cell Line

**Figure 4:**
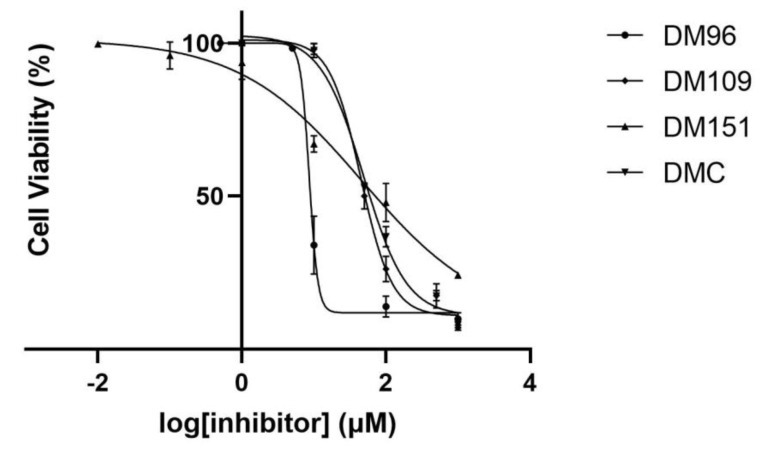
Concentration–response curves of the cytotoxicity of the most potent inhibitors DM96, DM109, DM151, and DMC against DU-145. The data were analyzed using the GraphPad Prism version 8.

The most potent inhibitors were also evaluated for their toxicity against DU-145 by means of an MTT assay, and the calculated IC_50_ values are summarized in Table 2, while the dose–response curves are presented in Figure 4. Based on the results, the strongest hGSTP1-1 inhibitor DM96 also exhibited the highest cytotoxic activity against DU-145 with an IC_50_ value of 8.60 ± 1.07 μΜ. The rest of the compounds induced moderate cytotoxicity of the same order of magnitude with IC_50_ values of 44.59 ± 1.08, 46.15 ± 3.68, and 48.52 ± 1.09 μΜ for DM109, DM151, and DMC respectively. The results of cytotoxicity for DM96 against DU-145 cells are comparable to the IC_50_ values reported in the literature for the parent molecule curcumin and its corresponding analogues in the same cell line and experimental conditions [50,81,82].

### 3.4. Inhibition Studies of Curcumin Analogues against Native GST Extracted from DU-145 Cells

Taking into consideration the observation that DU-145 cancer cells express high levels of the hGSTP1-1 enzyme, the inhibitory effect of DM96, DM109, DM151, and DMC was examined in DU-145 cell lysates [50]. Cell lysates were treated with the inhibitors at concentrations corresponding to the IC_50_ values of each compound (see Table 2). Consequently, the highest expected enzyme inhibition should be about 50%. The results (Table 3) confirmed that DM96, DM109, and DMC reduced the enzyme activity by almost 50%, as it is predicted by the corresponding IC_50_. The ability of the curcumin analogues to efficiently inhibit the native GST activity of DU-145 cells suggests that the compounds display satisfactory selectivity towards the native GST activity. Surprisingly, the DM151 derivative did not show any inhibition at all. This may be due to interactions with other cellular components in the lysate resulting in the lack of selectivity.

### 3.5. Circular Dichroism Study

**Figure 5:**
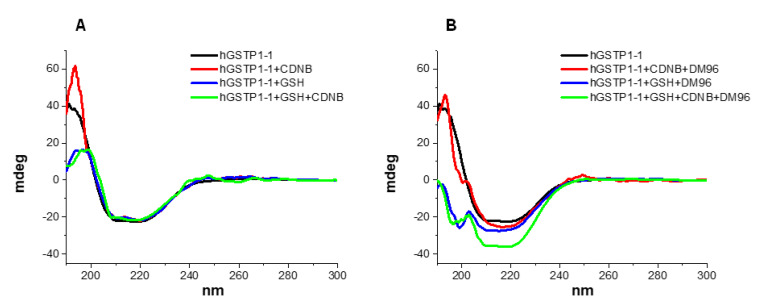
CD spectra of (**A**) hGSTP1-1 (0.1 mg/mL) isoenzyme in the absence (black line) or in the presence of substrate CDNB (1 mM; red line), GSH (2.5 mM; blue line), and mixture GSH + CDNB (2.5 mM + 1 mM, respectively; green line) and (**B**) hGSTP1-1 (0.1 mg/mL) isoenzyme in the absence (black line) or in the presence of inhibitor DM96 (5 μΜ) with substrate CDNB (1 mM; red line), GSH (2.5 mM; blue line) and mixture GSH + CDNB (2.5 mM + 1 mM, respectively; green line). Representative spectra from n = 3 independent experiments are presented.

Circular dichroism (CD) spectroscopy was employed to study the conformation and interactions of hGSTP1-1, and the spectra obtained are presented in Figure 5. The CD spectra of the isoenzyme show negative minima at 210 and 219 nm, as well as a positive maximum at 192 nm, conforming the general architecture (rich in α-helix) of cytosolic GSTs. An analysis of the secondary structure elements with the CDNN program gave a content of approximately 60% α-helix, 11% β-sheet, and 18% random coil, values that are in accordance with the total secondary structure content of the GST family that present about 48–59% α-helix and 8–10% β-strands [83]. Therefore, the CD data attest to the isolation of a well-folded protein from the expression and purification procedure, in agreement with the results on enzymatic activity (Section 3.1). The CD spectrum of the plain GSH solution under our conditions displays a broad negative peak at approximately 220 nm, while no CD signal is observed for the plain CDNB and plain DM96 in agreement with the fact that they are non-chiral molecules (Figure S3).

The addition of GSH (2.5 mM) in the solution of the enzyme causes a large drop in the intensity of the positive band at 192 nm with no apparent alterations in the characteristic negative bands of hGSTP1-1 (Figure 5A, blue line). An analysis with the CDNN program shows a decrease in the α-helix content and an increase in random coil conformation, indicative of structural changes. An abundance of information from spectroscopic kinetic, crystallographic, site-directed mutagenesis studies and computational dynamics approaches [83,84,85,86,87] show that GSH binding to the G-site of GSTs is associated with structural motions and transitions. These conformational changes are considered necessary in order to bring the GSH–GSTP1-1 complex to a catalytically active conformation and prepare the H-site for the binding of the hydrophobic co-substrate [87]. Despite the importance of this GSH binding in the catalytic mechanism of the enzyme, the CD spectra of the GSH–GST interaction are not—to the extent of our knowledge—available, and it appears that this is the first time that a CD spectrum of the interaction of GSH with GST is presented. The addition of the widely used CDNB substrate in the enzyme–GSH solution does not affect the CD spectrum, indicating that no significant changes in the secondary structure of the enzyme take place upon binding to CDNB (Figure 5A, green line) [83].

The presence of the strong inhibitor DM96 (5 μM) in the GSH–GSTP1-1 solution causes a great change in the CD spectrum of the GSH–GSTP1-1 mixture (Figure 5B, blue line). The positive band at 192 nm disappears, while a new negative band appears at 198 nm, indicating the increased presence of an unordered structure in the enzyme. The CD spectra show that the DM96 inhibitor brings about a distinct conformational effect, obviously involving more than plain binding to the hydrophobic substrate-binding site (H-site). The structural effect of DM96 on the enzyme (GSH–hGSTP1-1 complex) persists, and is even augmented, in the presence of CDNB, which is present in 200-fold excess compared to the inhibitor, supporting the notion that an alternative to the CDNB interaction mechanism is operating. Human GSTs are known to bind with a wide range of structurally diverse, non-substrate ligands at the dimer interface or in proximity to the hydrophobic H-site, resulting in the inhibition of GST catalytic function [88,89,90,91,92]. Even though the structural determinants of the binding are not known, it is generally accepted that the interaction of the non-substrate ligands with the enzyme restricts or alters the conformational dynamics of the enzyme, resulting in inhibition. In the case of the DM96 inhibitor, the strong conformational effect observed in the CD spectra correlates well with its inhibition potency. As DM96 shares to a great extent the potential for non-covalent interactions involving aromatic rings with the rest of the tested compounds, its distinct inhibitory activity may be associated with its hydrogen-bonding potential. The crystal data on hGSTP1-1 reveal that a double-stranded lattice of hydrogen-bonded water molecules connects the two active sites contributing to the structural and functional cooperativity of the two subunits [87]. Therefore, it appears plausible that the disruption of this hydrogen bond network by DM96 affects the structure stabilization of hGSTP1-1 and compromises its catalytic efficiency [93,94]. The deepening of the CD spectrum in the presence of CDNB may be indicative of the stabilization of the new enzyme conformation aided by the presence of the hydrophobic CDNB in solution. It is of interest that the presence of the very weak inhibitor DM62 in the enzyme mixture does not cause the conformational changes observed with DM96 (Figure S3, Supporting Information).

### 3.6. Molecular Docking of DM96, DM151, DM109, and DMC to hGSTP1-1

Molecular docking was used to explore the interactions of DM96, DM151, DM109, and DMC with hGSTP1-1. To generate initial complexes for subsequent molecular dynamics simulations, the four studied curcumin analogues were docked into the binding site pocket of hGSTP1-1 using the CANDOCK algorithm in conjunction with the generalized statistical scoring function RMR6 [57]. After the initial visual inspection, the binding modes of DM96, DM151, DM109, and DMC in complex with hGSTP1-1 were chosen based on the lowest docking score values presented in Table 4.

As can be inferred from Table 4, all investigated curcumin analogues exhibit satisfactory binding affinity to hGSTP1-1 (docking score values < −25 arb. units). The molecular docking results are also consistent with the obtained experimental data and support the hypothesis that the studied curcumin analogues exhibit an inhibitory effect on hGSTP1-1. Moreover, the docking results provided the first evaluation as to which curcumin analogues exhibit a stronger binding affinity (lower docking score value) to hGSTP1-1, namely DM96 and DM151, which exhibited a higher affinity to hGSTP1-1 than DM109 and DMC.

### 3.7. Molecular Dynamics Simulations of Studied Curcumin Analogues in Complex with hGSTP1-1

#### 3.7.1. RMSD and RMSF Analysis

**Figure 6:**
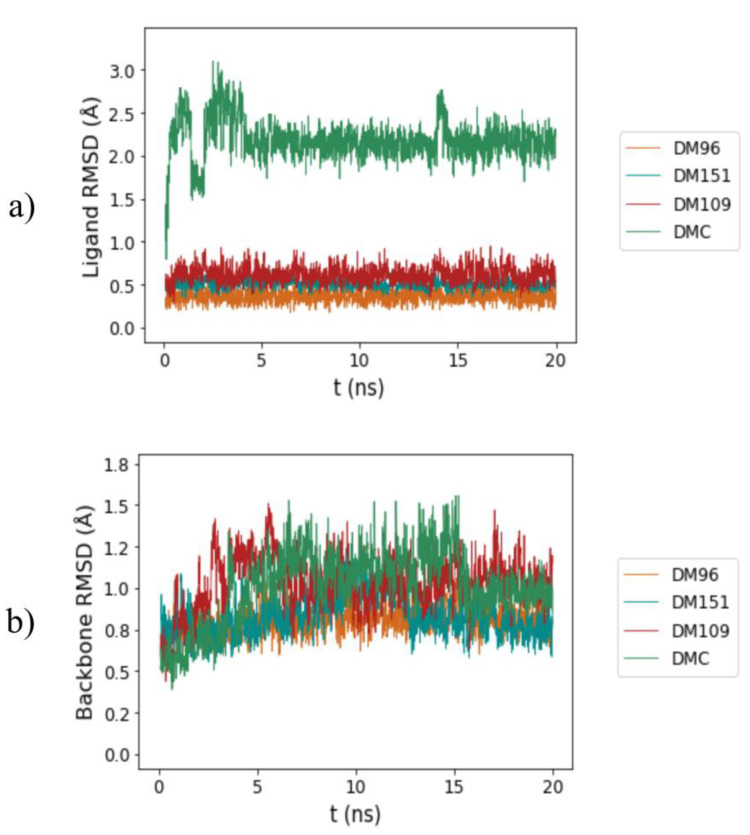
RMSD curves of: (**a**) curcumin derivative atomic positions, and (**b**) backbone atomic positions throughout 20 ns molecular dynamics simulations of hGSTP1-1/DM96 (red), hGSTP1-1/DM151 (orange), hGSTP1-1/DM109 (green), and hGSTP1-1/DMC (blue) complexes.

To investigate the stability of each complex, four independent 20 ns molecular dynamics simulations were performed for hGSTP1-1/DM96, hGSTP1-1/DM151, hGSTP1-1/DM106, and hGSTP1-1/DMC complexes obtained with molecular docking. The root mean square deviation (RMSD) of ligand and hGSTP1-1 backbone atoms during the entire production run was calculated to assess the conformational stability of the hGSTP1-1/curcumin derivative complexes. The RMSD values of each curcumin derivative were calculated with respect to its initial docked configuration after a translational and rotational fit of the active-site backbone atoms of hGSTP1-1. The RMSD values of the hGSTP1-1 backbone atoms with respect to their initial crystal structure positions were also calculated after a translational and rotational fit of the hGSTP1-1 backbone atoms. The average RMSD curves of the studied curcumin analogues and hGSTP1-1 backbone atoms are presented in Figure 6 under (a) and (b), respectively.

**Figure 7:**
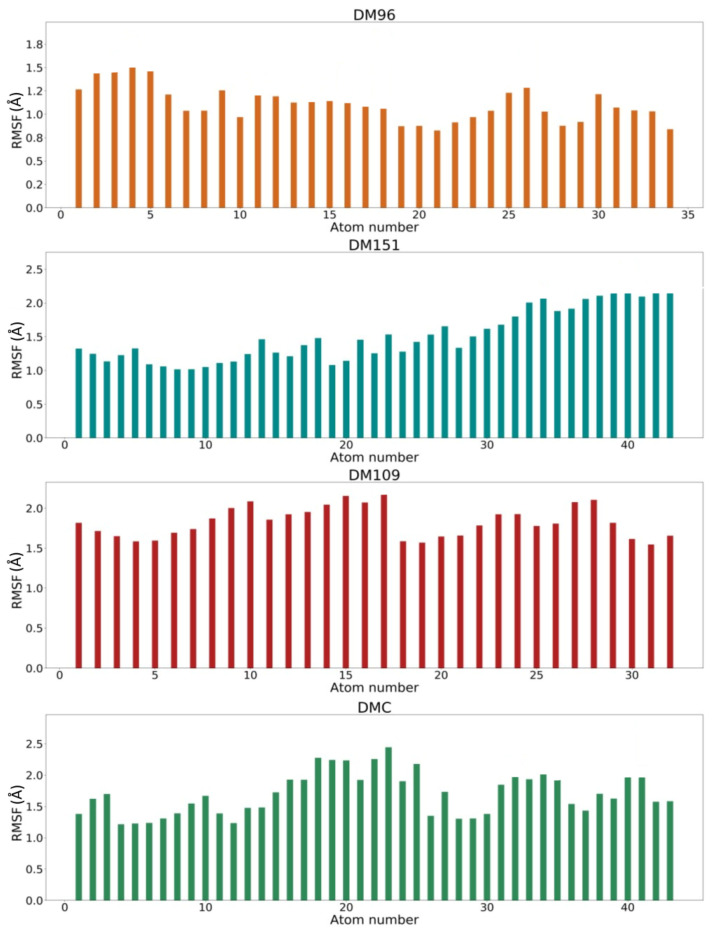
RMSF values of ligand atomic positions throughout molecular dynamics simulation production runs of hGSTP1-1 /DM96 (red), hGSTP1-1/DM151 (orange), hGSTP1-1/DM109 (green), and hGSTP1-1/DMC (blue) complexes.

Moreover, the root mean square fluctuations (RMSF) of the studied curcumin analogues in complex with hGSTP1-1 were calculated during the MD production runs. The RMSF values of the atomic positions of the four studied curcumin analogues are presented in Figure 7. The average ligand and backbone RMSD values together with the average ligand atomic positions and average backbone RMSF values of the four production runs are further collected in Table 5.

The stable RMSD curves presented in Figure 6a,b indicate that all simulated complexes were well-equilibrated, and that the equilibration protocol was appropriate for the hGSTP1-1 system. The RMSD curves for ligands DM96, DM151, DM109, and DMC stabilized at average values of 0.35 ± 0.02, 0.44 ± 0.03, 0.63 ± 0.03, and 2.10 ± 0.08 Å, respectively (Table 5).

The obtained results suggest that both the rigidity of the core as well as the number of phenol groups in the structure affect the stability of the atomic positions in the investigated curcumin analogues. The monocarbonyl derivative DM96 exhibited the lowest average RMSD values due to its rigid acetone core and two phenol groups. DM151 exhibited similar average RMSD values as DM96, presumably due to its large, rigid cyclohexanone core and four phenol groups, contributing to electrostatic attraction. The monocarbonyl derivate DM109 displayed slightly higher RMSD values, probably due to the absence of phenol groups in its structure. On the other hand, DMC exhibited the highest average RMSD values, due to its long diketone alkyl chain with a higher number of rotatable bonds, resulting in the least rigid structure. However, all average RMSD values indicate stable ligand poses for all the MD simulations (below 2.10 Å) [95]. Furthermore, from the RMSD curves for the backbone atoms with all four studied curcumin analogues presented in Figure 6b and Table 5, it can be observed that all the MD trajectories converged below 1.2 Å, once again indicating that equilibrium was achieved. The average RMSD backbone values for DM96, DM151, DM109, and DMC in complex with hGSTP1-1 were 0.76 ± 0.03, 0.85 ± 0.04, 1.01 ± 0.07, and 1.14 ± 0.06 Å, respectively.

The conformational stability of DM96, DM151, DM109, and DMC is indicated by average RMSF values of 1.25 ± 0.04, 1.55 ± 0.03, 1.81 ± 0.04, and 1.83 ± 0.05, respectively, which corresponds to previously discussed RMSD values of the ligands (Table 5). In addition, from Figure 7, it can be observed that the middle part of DMC is more flexible than the middle parts of DM96, DM151, and DM109, mainly due to its less rigid diketone core. As can be observed from Table 5, the average RMSF values remain below 2 Å, suggesting that the conformations of all curcumin analogues as well as hGSTP1-1 amino acid residues remain close to their initial structures.

#### 3.7.2. Binding Patterns of DM96, DM151, DM109, and DMC to hGSTP1-1

**Figure 8:**
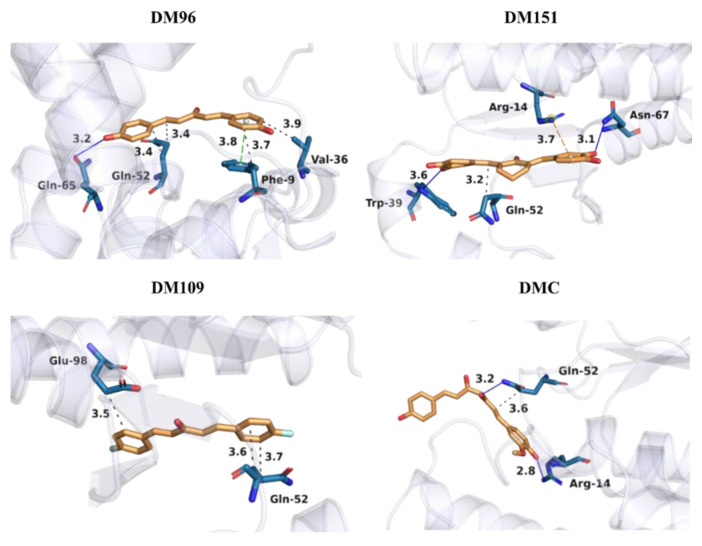
Binding modes of DM96, DM151, DM109, and DMC at the active site of hGSTP1-1. Carbon atoms of the studied curcumin derivatives are presented in orange, while carbon atoms of hGSTP1-1 amino-acid residues are depicted in light blue color. Oxygen atoms are red, nitrogen atoms dark blue, and fluorine atoms green. Hydrophobic interactions are presented with gray dashed lines and hydrogen bonds with dark blue lines. All distances represent averages over all MD production run snapshots for each complex and are provided in Å. Hydrogen atoms are omitted for reasons of clarity.

The detailed interactions between the studied curcumin analogues and hGSTP1-1 were further evaluated using the Protein Ligand Interaction Profiler (PLIP) [96]. The important interactions between the four investigated curcumin analogues and key binding amino-acid residues of hGSTP1-1 are presented in Figure 8. The occurrences of the most important hydrogen bonds between hGSTP1-1 and the studied curcumin analogues were also monitored according to the geometric criterium used in the PLIP algorithm.

From the binding modes in Figure 8, it can be deduced that all studied curcumin analogues form hydrophobic interactions with residue Gln52—for example, DM96 at distances 3.41 and 3.44 Å, DM151 at 3.20 Å, DM109 at distances 3.59 and 3.71 Å, and DMC at 3.59 Å. DM96 is additionally stabilized through hydrophobic interactions with nearby residues Phe9 and Val36 at distances of 3.70 and 3.92, respectively, as well as through a π–π stacking interaction with Phe8 at a distance of 3.77 Å. Moreover, DM151 is additionally stabilized by a π–cation interaction with Arg14 (3.95 Å), while a strong hydrophobic interaction with Glu98 was observed in the case of DM109 (3.45 Å). In addition, DM96 forms a hydrogen bond with the nearby residue Gln65 (2.90 Å, 85.16% occurrence), DM151 with residues Trp39 (3.07 Å, 94.21% occurrence) and Asn67 (2.24 Å, 81.89% occurrence), and DMC with residues Gln52 (2.45 Å, 80.84% occurrence) as well as Arg14, (2.05 Å, 96% occurrence), while DM109 does not form any hydrogen bonds due to the absence of phenolic groups. The hydrophobic interactions, therefore, play a vital role in stabilizing all four investigated inhibitors in the active pocket, while hydrogen bonds contribute to a lesser extent. It can be observed that similar amino acid residues from the G active site play an important part in the binding of the investigated curcumin analogues to hGSTP1-1, which confirms that all four analogues occupy the same active site.

### 3.8. Binding-Free-Energy Calculations with the LIE and LRA Methods

The binding-free-energy calculations of the studied curcumin analogues to the binding pocket of hGSTP1-1 were also performed. The van der Waals (vdW) and electrostatic (ele) non-bonded interactions were calculated for all four investigated curcumin analogues with LIE and LRA methods to compare their binding affinity.

The binding free energies of DM96, DM151, DM109, and DMC to hGSTP1-1, calculated by using preoptimized values of the empirical coefficients 0.5 for α and 1.043 for β [78,79] of the LIE and LRA Equations (1) and (2) (see Section 2.2), are collected in Table 6. For comparison, the experimental binding free energies calculated from the experimentally measured values of Ki and K_i΄_ for the studied curcumin analogues are also provided in Table 2.

In Table 6, it can be observed that the LIE and LRA methods predicted more negative binding free energies for the DM96/hGSTP1-1 complex (−7.83 ± 0.27 kcal/mol and −8.11 ± 0.28 kcal/mol, respectively) than for the DM151/hGSTP1-1 (−7.49 ± 0.50 kcal/mol and −7.73 ± 0.51 kcal/mol, respectively), DM109/hGSTP1-1 (−5.38 ± 0.07 kcal/mol and −5.60 ± 0.08 kcal/mol, respectively), and DMC/hGSTP1-1 (−4.88 ± 0.20 kcal/mol and −5.09 ± 0.21 kcal/mol, respectively) complexes, which is in agreement with the order of the docking score values from Table 4 as well as corresponds to the experimentally obtained binding free energies presented in Table 2.

The binding free energies revealed that non-polar van der Waals interactions through shape complementarity represent the main driving force for binding and the inhibitory activity of DM96 (−40.35 ± 0.44 kcal/mol) and DM109 (−34.49 ± 0.07 kcal/mol) binding to hGSTP1-1. On the contrary, hydrogen bonding through electrostatic interactions plays the main role in DM151’s (−47.52 ± 0.43 kcal/mol) and DMC’s (−55.11 ± 0.29 kcal/mol) inhibitory activities, while van der Waals interactions play a minor role (−39.88 ± 0.54 kcal/mol and −37.08 ± 0.06 kcal/mol, respectively). Moreover, electrostatic interactions also significantly contributed to DM96 binding (−36.05 ± 0.1 kcal/mol), while the least negative electrostatic contribution was observed in the case of DMC (−21.41 ± 0.05 kcal/mol) due to the absence of phenolic groups in its structure.

In addition, lower average protein–ligand interaction energies in complex when compared to free analogues in water also indicate favorable van der Waals and electrostatic contributions to binding between the studied curcumin analogues and hGSTP1-1. The binding-site dipoles associated with polar groups and ionized residues are already partially oriented towards all bound ligands with all their partial atomic charges set to zero, which is indicated by favorable preorganized electrostatic interactions between hGSTP1-1 and the investigated curcumin analogues ($\langle V_{ele}^{L-P}\rangle_{0}$ < 0). It can be concluded that van der Waals/hydrophobic interactions play a major role in the DM96 and DM109 mechanisms of inhibition, while electrostatic/hydrogen interactions mainly contribute to the binding of DM151 and DMC to hGSTP1-1.

## 4. Conclusions

Anti-cancer drug resistance is a complex process resulting from different defensive mechanisms in cancer cells. Numerous published studies have established the role of hGSTP1-1 in MDR and have connected its cancer drug-detoxifying ability with its response to chemotherapy. In the present work, two curcuminoids and eleven curcumin analogues were synthesized and their function towards the hGSTP1-1 and DU-145 cancer cell line were assessed. The computational and experimental results of the study allowed the identification of the monocarbonyl curcumin derivative DM96 as the most promising compound with the higher inhibition potency among four hGSTP1-1 inhibitors (DM96, DM151, DM109, and DMC).

The binding free energies calculated by the LIE and LRA methods predicted more negative binding free energy for the hGSTP1-1/DM96 complex than for the hGSTP1-1/DM151, hGSTP1-1/DM109, and hGSTP1-1/DMC complexes, a result which is fully consistent with the inhibitory potency order predicted by molecular docking, and in agreement with the experimental binding free energies obtained with GSH and CDNB substrates. It was also revealed that the van der Waals component through shape complementarity represents an important driving force for the binding and inhibitory activity of the most potent hGSTP1-1 inhibitor DM96 as well as for the inhibitory activity of DM109, while the electrostatic component through hydrogen-bonding plays a crucial role in DM151 and DMC binding. Moreover, all studied analogues form hydrophobic interactions with residue Gln52, and hydrogen bonds with nearby residues Gln65 and Asn67, while DM96, the most potent inhibitor, is additionally stabilized through hydrophobic interactions with the nearby residues Phe9 and Val36 as well as through a π–π stacking interaction with Phe9. The interactions with amino acid residues from the G-site in the N-terminal region of hGSTP1-1 are, therefore, considered to be the most important for the binding of the studied curcumin analogues.

Taking into consideration the observation that DM96 exhibits both high inhibition potency and cytotoxicity, it can be concluded that this monocarbonyl curcumin derivative could be a useful bifunctional candidate molecule able to contribute simultaneously to both the chemosensitization of the cancer cells as well as to cytotoxicity against cancer cells.

## Figures and Tables

**Table 1:** Screening of the inhibition potency of the curcuminoids (curcumin and DMC) and the curcumin analogues against hGSTP1-1 isoenzyme.

Compound Code	Molecular Weight	Enzyme Inhibition (%)
**Curcumin**	368.38	53.00 ± 1.98
**DMC**	338.35	94.56 ± 0.25
**DM15**	274.36	66.23 ± 2.57
**DM46**	334.40	8.50 ± 2.13
**DM57**	394.46	16.75 ± 3.61
**DM62**	310.34	8.40 ± 0.36
**DM95**	354.39	44.18 ± 3.06
**DM96**	266.29	86.99 ± 1.88
**DM100**	366.41	40.00 ± 1.84
**DM101**	326.34	72.28 ± 1.91
**DM109**	270.27	79.06 ± 2.54
**DM148**	298.29	54.80 ± 0.52
**DM151**	338.35	88.18 ± 3.65

**Table 2:** Inhibition constants and IC_50_ values obtained by kinetic inhibition and cytotoxicity studies of the most potent inhibitors DM96, DM151, DM109, and Curcumin II. The *in-silico*-determined binding free energies (kcal/mol) are also included.

Inhibitor	IC_50_ against DU-145 (μΜ)	IC_50_ against hGSTP1-1 (μΜ)	Variable Substrate	Type of Inhibition	Inhibition Constants (μΜ)	Experimental Binding Free Energies (kcal/mol)
**DM96**	8.60 ± 1.07	5.45 ± 1.08	**CDNB**	Purely mixed	K_i_ = 3.67 ± 0.35 K_i΄_ = 4.97 ± 2.86	ΔGexp = −7.71 ΔG′exp = −7.52
			**GSH**	Partial mixed	K_i_ = 3.69 ± 1.00 K_i΄_ = 1.45 ± 0.43	ΔGexp = −7.71 ΔG′exp = −8.29
**DM151**	44.59 ± 1.08	11.17 ± 1.03	**CDNB**	Purely non-competitive	K_i_ = 9.55 ± 2.36	ΔGexp = −7.12
			**GSH**	Purely non-competitive	K_i_ = 5.79 ± 1.21	ΔGexp = −7.43
**DM109**	46.15 ± 3.68	19.53 ± 1.04	**CDNB**	Purely non-competitive	K_i_ = 20.12 ± 5.27	ΔGexp = −6.66
			**GSH**	Purely non-competitive	K_i_ = 35.12 ± 5.69	ΔGexp = −5.96
**Curcumin II**	48.52 ± 1.09	37.72 ± 1.02	**CDNB**	Partially mixed	Κ_i_ = 3.99 ± 1.80 K_i΄_ = 2.36 ± 1.37	ΔGexp = −7.66 ΔG′exp = −7.98
			**GSH**	Partially mixed	Κ_i_ = 68.02 ± 14.60 K_i΄_ = 52.83 ± 12.25	ΔGexp = −5.91 ΔG′exp = −6.07

**Table 3:** Experimental data related to the potential of the most potent inhibitors (DM96, DM109, DM151, and DMC) to inhibit GST activity in DU-145 cell lysate.

Compound Code	Enzyme Inhibition (%)
**DM96**	41.10 ± 1.99
**DM109**	50.16 ± 0.76
**DM151**	No inhibition was observed
**DMC**	48.30 ± 2.51

**Table 4:** Docking score values of the best-scored hGSTP1-1-curcumin analogur complexes obtained with the CANDOCK algorithm in conjunction with scoring function RMR6.

Curcumin Derivative	Docking Score Values (Arbitrary Units)
**DM96**	−40.63
**DM151**	−36.99
**DM109**	−31.30
**DMC**	−25.39

**Table 5:** Average RMSD values of ligand and backbone atomic positions together with average RMSF values of ligand and backbone atomic positions throughout four independent 20 ns molecular dynamics simulation production runs of hGSTP1-1/DM96, hGSTP1-1/DM151, hGSTP1-1/DM109, and hGSTP1-1/DMC complexes. The average values of all four production runs are presented as mean ± standard deviation.

Curcumin Derivative	Average Ligand RMSD (Å)	Average Backbone RMSD (Å)	Average Ligand RMSF (Å)	Average Backbone RMSF (Å)
**DM96**	0.35 ± 0.02	0.76 ± 0.03	1.25 ± 0.04	0.77 ± 0.02
**DM151**	0.44 ± 0.03	0.85 ± 0.04	1.55 ± 0.03	0.79 ± 0.01
**DM109**	0.63 ± 0.03	1.01 ± 0.07	1.81 ± 0.04	0.86 ± 0.06
**DMC**	2.10 ± 0.08	1.14 ± 0.06	1.83 ± 0.05	0.90 ± 0.07

**Table 6:** The average electrostatic (ele) and van der Waals (vdW) non-bonded interactions of DM96, DM151, DM109, and DMC in water (the free state W) as well as in complex with hGSTP1-1 (the bound state P) along with the corresponding binding free energies.

Energies	$\langle V_{vdW}^{L-P}\rangle$ (kcal/mol)	$\langle V_{vdW}^{L-W}\rangle$ (kcal/mol)	$\langle V_{ele}^{L-P}\rangle$ (kcal/mol)	$\langle V_{ele}^{L-W}\rangle$ (kcal/mol)	$\langle V_{ele}^{L-P}\rangle_{0}$ (kcal/mol)	$\Delta G_{binding}^{L-P}$ ** (kcal/mol)
**DM96**						
LIE average *	−40.52 ± 0.44	−23.49 ± 0.03	−36.05 ± 0.1	−36.7 ± 0.04	/	−7.83 ± 0.27
LRA average *	−40.52 ± 0.44	−23.49 ± 0.03	−36.05 ± 0.1	−36.7 ± 0.04	−0.28 ± 0.02	−8.11 ± 0.28
**DM151**						
LIE average *	−39.88 ± 0.54	−28.23 ± 0.04	−47.52 ± 0.43	−45.91 ± 0.19	/	−7.49 ± 0.50
LRA average *	−39.88 ± 0.54	−28.23 ± 0.04	−47.52 ± 0.43	−45.91 ± 0.19	−0.23 ± 0.01	−7.73 ± 0.51
**DM109**						
LIE average *	−34.49 ± 0.07	−24.83 ± 0.01	−21.41 ± 0.05	−20.89 ± 0.01	/	−5.38 ± 0.07
LRA average *	−34.49 ± 0.07	−24.83 ± 0.01	−21.41 ± 0.05	−20.89 ± 0.01	−0.22 ± 0.01	−5.60 ± 0.08
**DMC**						
LIE average *	−37.08 ± 0.06	−28.80 ± 0.02	−55.11 ± 0.29	−54.4 ± 0.12	/	−4.88 ± 0.20
LRA average *	−37.08 ± 0.06	−28.80 ± 0.02	−55.11 ± 0.29	−54.4 ± 0.12	−0.21 ± 0,01	−5.09 ± 0.21

* The average values of four independent production runs are presented as mean ± standard deviation. Standard deviations were calculated from corresponding mean contributions obtained in four independent 20 ns production runs of MD simulations. ** Binding free energies were calculated with preoptimized values of α = 0.5 and β = 1.043.

## Data Availability

All relevant data are included in the article and/or its Supplementary Information files.

## Supplementary Materials

The following supporting information can be downloaded at: https://www.mdpi.com/article/10.3390/antiox12010063/s1, Experimental Part, and Figure S1 Kinetics inhibition studies of DM151, DM109 and Curcumin II using CDNB as variable substrate, Figure S2 Kinetics inhibition studies of DM151, DM109 and Curcumin II using GSH as variable substrate, Figure S3 CD spectra. References [97,98,99,100,101,102] are cited in the supplementary materials.

## Abbreviations

BDMC	Bisdemethoxycurcumin
Bis-epoxirane	1,4-butanediol diglycidyl ether
CDNB	1-chloro-2,4-dinitrobenzene
DM100	2,6-bis(3-methoxy-4-hydroxy-benzylidene)cyclohexanone
DM101	(1E,4E)-1,5-bis-(3-methoxy-4-hydroxy-phenyl)penta-1,4-dien-3-one
DM109	(1E,4E)-1,5-bis(4-fluorophenyl)penta-1,4-dien-3-one
DM148	(1E,4E)-1,5-bis(3,4-dihydroxyphenyl)penta-1,4-dien-3-one
DM15	2,6-dibenzylidenecyclohexanone
DM15	2,6-dibenzylidenecyclohexanone
DM151	2,6-bis(3,4-dihydroxybenzylidene)cyclohexanone
DM46	2,6-bid(4-methoxybenzylidene)cyclohexanone
DM57	2,6-bis(3,4-dihydroxybenzylidene)cyclohexanone
DM62	(2E,6E)-2,6-bis(4-fluorobenzylidene)cyclohexanone
DM95	(1E,4E)-1,5-bis(3,4-dimethoxyphenyl)penta-1,4-dien-3-one
DM96	1,5-bis(4-hydroxyphenyl)penta-1,4-dien-3-one
DMC	Demethoxycurcumin
DMSO	Dimethyl sulfoxide
*E. coli*	*Escherichia coli*
EDTA	Ethylenediaminetetraacetic acid
ESP	Electrostatic potential
FBS	Fetal bovine serum
GAFF	General AMBER force field
GSH	Glutathione
GSTs	Glutathione transferases
IPTG	Isopropyl-β-D-thiogalactopyranoside
LIE	Linear interaction energy
LRA	Linear response approximation
LRF	Local reaction field
MD	Molecular dynamics simulations
MTT	(3-[4,5-dimethylthiazol-2-yl]-2,5-diphenyl-tetrazolium bromide)
NaCl	Sodium chloride
NCSR	National Center for Scientific Research
PLIP	Protein Ligand Interaction Profiler
RESP	Restricted electrostatic charge fitting procedure
RMR6	Radial-mean-reduced scoring function at a cutoff radius of 6 Å from each atom of the ligand
RMSD	Root-mean-square deviation
RMSF	Root-mean-square fluctuation
SCAAS	Surface constraint all-atom solvent
SDS	Sodium dodecyl sulfate
SDS-PAGE	Sodium dodecyl sulphate–polyacrylamide gel electrophoresis
