# Supplementary material for: Monocarbonyl Curcumin Analogues as Potent Inhibitors against Human Glutathione Transferase P1-1
Source: Antioxidants (Basel). 2022 Dec 28;12(1):63. doi: 10.3390/antiox12010063 (PMC9854774; doi:10.3390/antiox12010063)
Supplement: Supplementary file 1 [file antioxidants-12-00063-s001.zip › antioxidants-2093971-supplementary.pdf]

Supporting Information

1. Experimental Part

1.1. General information

All reagents were purchased from Sigma-Aldrich, Alfa Aesar and TCI and used without further purification. NMR spectra were recorded with a Bruker Avance 500 MHz spectrometer (Bruker, Rheinstetten, Germany) operating at 500 MHz (^1H). Chemical shifts are reported in ppm relative to DMSO-d_6 (^1H: δ = 2.50 ppm). Syntheses of the following compounds were achieved using previously published protocols from our group without -or with slight- modifications.

1.2. Synthesis of curcumin derivatives

1.2.1. Procedure for the synthesis of dicarbonyl curcumins

Boron trioxide (0.35 g, 5.0 mmol), tributyl borate (10.8 mL, 40 mmol), acetylacetonate (1.03 mL, 10.0 mmol), vanillin (1.52 g, 10 mmol) and 4-hydroxybenzaldehyde (1.22 g, 10 mmol) added in a 50 mL round-bottom flask. The mixture was stirred at 90 °C for 30 min. To this mixture, *n*-butylamine (0.4 mL, 4.0 mmol) was added dropwise over 30 min at 70 °C. Then, the mixture was stirred at 100 °C for 90 min and at 70 °C for 24 h. Hydrochloric acid (30 mL, 1 M) was added and the mixture was stirred at 60 °C for 2 h. The mixture was extracted with ethyl acetate (3 × 30 mL). The combined organic extracts were washed with brine, dried with Na_2SO_4, and concentrated under vacuum. The oily product was purified two times by flash column chromatography. The first was done using $CHCl_3$:MeOH at ratio 100/0 to 95/5. Curcumin and DMC were isolated by flash column chromatography using $CHCl_3$:MeOH at ratios 99.3/0.7 and 98/2, respectively.

<u>1*E*,6*E*)-1,7-bis (4-hydroxy-3-methoxyphenyl) hepta-1,6-diene-3,5-dione (**Curcumin**)</u> [1]

Orange solid, Yield: 1.22 g (33%); ^1H NMR (500 MHz, DMSO-d_6): δ 3.83 (s, 6H), 6.06 (s, 2H), 6.76 (d, J = 15.8 Hz, 2H), 6.82 (d, J = 8.0 Hz, 2H), 7.15 (d, J = 8.0 Hz, 2H), 7.32 (s, 2H), 7.55 (d, J = 15.8 Hz, 2H), 9.65 (s, 4H).

(1E,6E)-1-(4-hydroxy-3-methoxyphenyl)-7-(4-hydroxyphenyl)hepta-1,6-diene-3,5-dione (**DMC**) [1]

Orange solid, Yield: 512 mg (16%); ^{1}H NMR (500 MHz, DMSO-d_6): δ 3.83 (s, 3H), 6.04 (s, 1H), 6.70 (d, *J* = 15.8 Hz, 1H), 6.75 (d, *J* = 15.8 Hz, 1H), 6.82 (m, 3H), 7.14 (d, *J* = 7.8 Hz, 1H), 7.32 (s, 1H), 7.52–7.57 (m, 4H), 9.68 (br, 1H), 10.05 (br, 1H).

1.2.2. General procedure for the synthesis of monocarbonyl curcuminoids without hydroxyl groups

Acetone (0.73 mL, 10 mmol, for compound DM95) or cyclohexanone (0.98 g, 10 mmol, for compounds DM57, DM46 and DM62 was added to a solution of veratraldehyde (3.32 g, 20 mmol, for compounds DM95 and DM57) or p-anisaldehyde (2.72 g, 20 mmol, for compound DM46) or 4-fluorobenzaldehyde (2.48 g, 20 mmol, for compound DM62) in EtOH (10 mL). To this mixture, a dispersion of NaOH (5 g, 125 mmol) in EtOH (40 mL) was added and the resulting mixture was stirred at r.t. for 3 h. Water (30 mL) was added to the mixture and the formed precipitate was filtered, washed with water, and recrystallized from EtOH.

(1*E*,4*E*)-1,5-bis (3,4-dimethoxyphenyl) penta-1,4-dien-3-one (**DM95**) [1]

Yellow needles, Yield: 2.84 mg (72%); ^{1}H NMR (500 MHz, DMSO-d_6): δ 3.82 (s, 6H), 3.84 (s, 6H), 7.03 (d, *J* = 8.3 Hz, 2H), 7.23 (d, *J* = 15.9 Hz, 2H), 7.33 (d, *J* = 8.3 Hz, 2H), 7.41 (s, 2H), 7.70 (d, *J* = 15.9 Hz, 2H).

2,6-bis((*E*)-4-methoxybenzylidene)cyclohexan-1-one (**DM46**) [2]

Yellow needles, Yield: 2.42 g (72%); ^{1}H NMR (500 MHz, DMSO-d_6): δ 1.70-1.75 (m, 2H), 2.86-2.89 (m, 4H), 3.81 (s, 6H), 7.02 (d, *J* = 8.2 Hz, 2H), 7.52 (d, *J* = 8.2 Hz, 2H), 7.59 (s, 2H).

2,6-bis((*E*)-3,4-dimethoxybenzylidene)cyclohexan-1-one (**DM57**) [3]

Yellow needles, Yield: 3.04 g (77%); ^{1}H NMR (500 MHz, DMSO-d_6): δ 1.71-1.75 (m, 2H), 2.90-2.92 (m, 4H) 3.80 (s, 12H), 7.03 (d, *J* = 8.6 Hz, 2H), 7.12-7.16 (m, 4H), 7.59 (s, 2H).

2,6-bis((*E*)-4-fluorobenzylidene)cyclohexan-1-one (**DM62**) [4]

Light yellow solid; Yield: 2.20 g (70%); ^{1}H NMR (500 MHz, DMSO-d_6): δ 1.69-1.74 (m, 2H), 2.86-2.89 (m, 4H), 7.28 (d, *J* = 8.2 Hz, 2H), 7.30 (d, *J* = 8.2 Hz, 2H), 7.58-7.63 (m, *4*H).

1.2.3. General procedure for the synthesis of dihydroxy monocarbonyl curcuminoids

Acetone (0.73 mL, 10 mmol, for DM101 and DM96) or cyclohexanone (0.98 g, 10 mmol, for DM100 and DM15) was added to a solution of 4-hydroxybenzaldehyde (2.44 g, 20 mmol, for DM96 and DM15) or vanillin (3.04 g, 20 mmol, for compounds DM100 and DM101) in ethanol (3 mL). To this mixture, concentrated hydrochloric acid (0.2 mL) was added and the solution was stirred at r.t. for 24 h. Then, the solution was concentrated under vacuum to half volume, poured into ice-cold water (40 mL), and 1% aqueous KOH solution was added until pH 6-7. Then, the precipitate was filtered and washed with water (×2) and hot water (×2) for DM100, DM96 and DM15 or only with water (<35 °C, ×2) for DM101.

(1*E*,4*E*)-1,5-bis(4-hydroxy-3-methoxyphenyl)penta-1,4-dien-3-one (**DM101**) [3]

Orange powder; Yield: 2.15 g (66%), ^{1}H NMR (500 MHz, DMSO-d_6): δ 3.85 (s, 6H), 6.83 (d, J = 8.2 Hz, 2H), 7.15 (d, J = 16.0 Hz, 2H), 7.20 (d, J = 8.2 Hz, 2H), 7.37 (s, 2H), 7.65 (d, J = 16.0 Hz, 2H), 9.66 (s, 2H).

2,6-bis((*E*)-4-hydroxy-3-methoxybenzylidene)cyclohexan-1-one (**DM100**) [3]

Dark green powder; Yield: 2.55 g (70%), ^{1}H NMR (500 MHz, DMSO-d_6): δ 1.70-1.75 (m, 2H), 2.88-2.91 (m, 4H) 3.81 (s, 6H), 6.85 (d, J = 8.2 Hz, 2H), 7.03 (d, J = 8.2 Hz, 2H), 7.11 (s, 2H), 7.56 (s, 2H), 9.53 (s, 2H).

(1*E*,4*E*)-1,5-bis(4-hydroxyphenyl)penta-1,4-dien-3-one (**DM96**) [5]

Light green powder, Yield: 1.71 g (64%), ^{1}H NMR (500 MHz, DMSO-d_6) δ: 6.83 (d, J = 8.2 Hz, 4H), 7.10 (d, J = 16.0 Hz, 2H), 7.62 (d, J = 8.6 Hz, 2H), 7.66 (d, J = 8.6 Hz, 2H), 10.03 (s, 2H).

2,6-bis((*E*)-4-hydroxybenzylidene)cyclohexan-1-one (**DM15**) [6]

Light green powder; Yield: 2.23 g (73%), ^{1}H NMR (500 MHz, DMSO-d_6): δ 1.67-1.75 (m, 2H), 2.82-2.88 (m, 4H), 6.84 (d, *J* = 8.4 Hz, 4H), 7.41 (d, *J* = 8.4 Hz, 4H), 7.54 (s, 2H), 9.93 (s, 2H).

1.2.4. General procedure for the synthesis of tetrahydroxy monocarbonyl curcuminoids

DM95 (133 mg, 0.5 mmol, for DM148) or DM57 (153 mg, 0.5 mmol, for DM151) was dissolved in anhydrous DCM (10 mL) in a two-necked round bottom flask under N_2. The solution was cooled to -20 °C using ice/NaCl bath. Boron tribromide (0.23 mL, 2.5 mmol) was injected carefully with a syringe. The reaction mixture was stirred for 1 h at -20 °C, then for 1 h at 0 °C and for 1 h at room temperature. Then, ice-cold water was poured into the mixture, and the flask was shaken for a few minutes. The resulting precipitate was filtered and washed with small amounts of water. An additional amount of product was recovered from the filtrate after extraction with EtOAc. Compounds were purified by flash column chromatography using $CHCl_3$:MeOH = 95/5 to 90/10.

(1*E*,4*E*)-1,5-bis(3,4-dihydroxyphenyl)penta-1,4-dien-3-one (**DM148**) [3]

Green solid; Yield: 79 mg (53%), ^{1}H NMR (500 MHz, DMSO-d_6): 6.79 (d, *J* = 8.2 Hz, 2H), 6.99 (d, *J* = 16.0 Hz, 2H), 7.07 (d, *J* = 8.2 Hz, 2H), 7.14 (s, 2H), 7.56 (d, *J* = 16.0 Hz, 2H), 9.38 (br, 4H).

(2*E*,6*E*)-2,6-bis(3,4-dihydroxybenzylidene)cyclohexanone (**DM151)** [3]

Green solid; Yield: 120 mg (71%), ^{1}H NMR (500 MHz, DMSO-d_6): 1.69-1.74 (m, 2H), 2.83-2.86 (m, 4H), 6.80 (d, *J* = 8.2 Hz, 2H), 6.87 (d, *J* = 8.2 Hz, 2H), 6.98 (s, 2H), 7.45 (s, 2H), 9.13 (s, 1H), 9.44 (s, 1H).

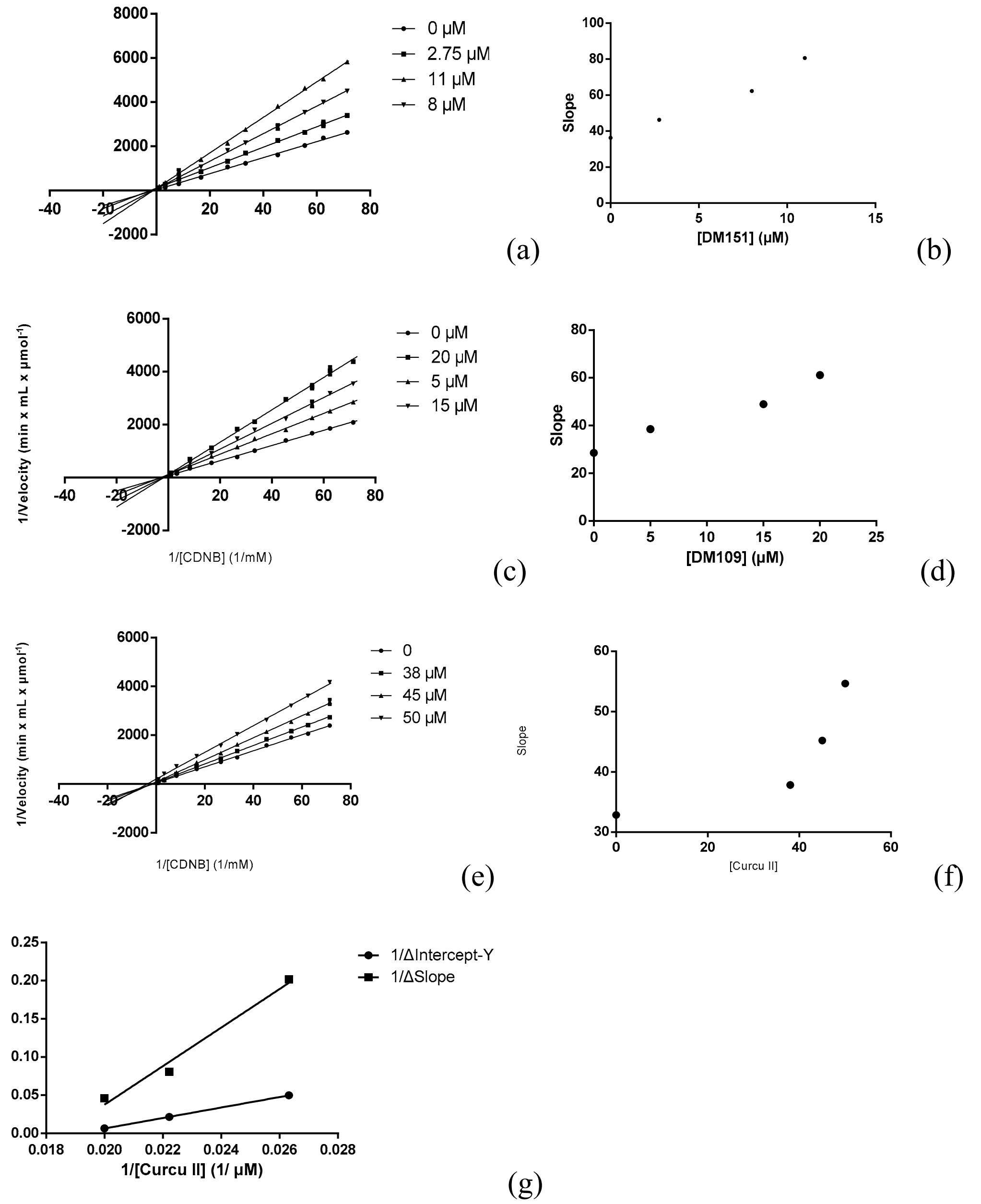

Figure S1. Kinetics inhibition studies. Lineweaver–Burk graphs, presenting the hGSTP1-1 isoenzyme initial velocities versus the concentrations of CDNB (18 – 980 µM), at different constant concentrations of **(a)** DM151 (0, 2.75, 8 and 11 µM), **(c)** DM109 (0, 5, 15 and 20) and **(e)** Curcumin II (0, 38, 45 and 50 µM). The lines of the graph indicate that DM151 and DM109 have a profile of purely non-competitive inhibitor and Curcumin II a partially mixed towards CDNB. Secondary plots for **(b)** DM151, **(d)** DM109 and **(f)** Curcumin II resulting from the representation of the slopes as a function of the inhibitor concentrations. **(g)** Tertiary plot of Curcumin II that depicts the 1/ΔIntercept -Y and 1/Δslope as a function of the 1/[inhibitor]. The graphs were created using the computer program GraphPad Prism version 8.

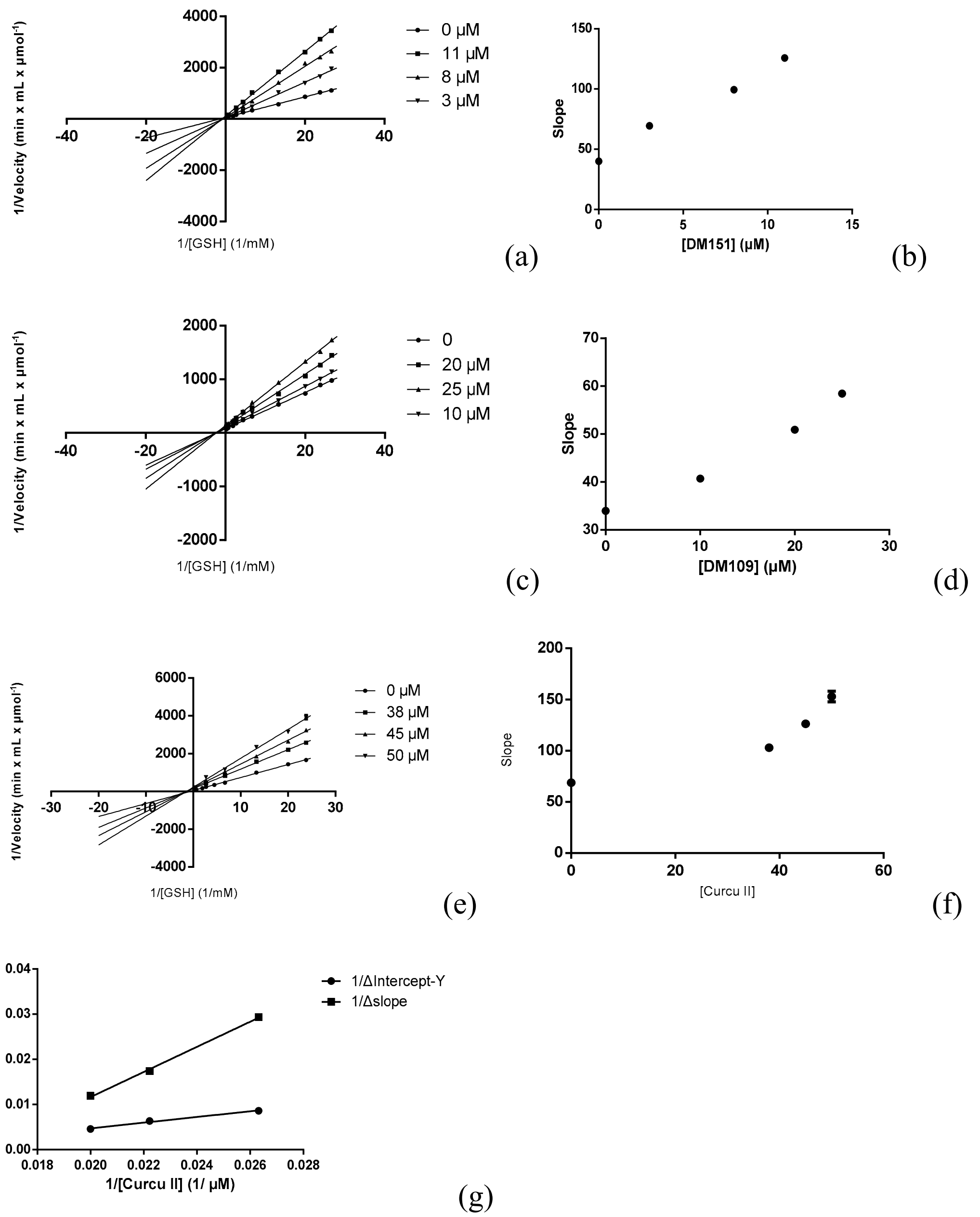

Figure S2. Kinetics inhibition studies. Lineweaver–Burk graphs, presenting the hGSTP1-1 isoenzyme initial velocities versus the concentrations of GSH (37.5 – 3750 μM), at different constant concentrations of **(a)** DM151 (0, 3, 8 and 11 μM), **(c)** DM109 (0, 5, 15 and 20) and **(e)** Curcumin II (0, 38, 45 and 50 μM). The lines of the graph indicate that DM151 and DM109 have a profile of purely non-competitive inhibitor and Curcumin II a partially mixed towards CDNB. Secondary plots for **(b)** DM151, **(d)** DM109 and **(f)** Curcumin II resulting from the representation of the slopes as a function of the inhibitor concentrations. **(g)** Tertiary plot of Curcumin II that depicts the 1/ΔIntercept -Y and 1/Δslope as a function of the 1/[inhibitor]. The graphs were created using the computer program GraphPad Prism version 8.

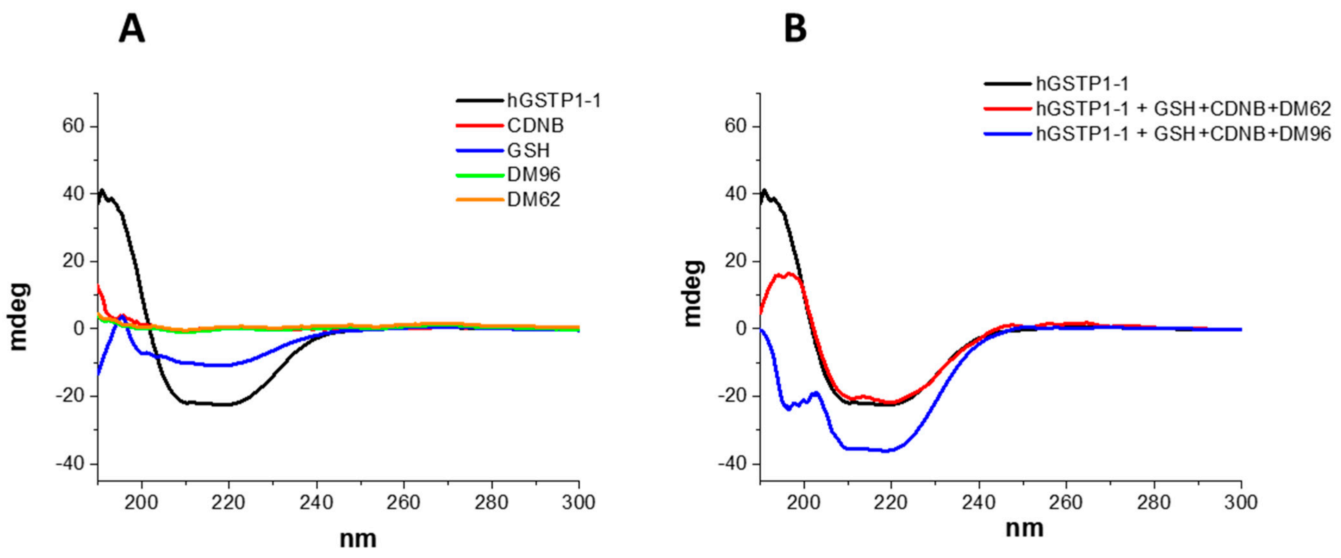

Figure S3. CD spectra of (**A**) plain solutions of hGSTP1-1 (0.1mg·ml^{-1}, black line), substrate CDNB (1 mM, red line), GSH (2.5 mM, blue line), DM96 (5 µM, green line) and DM62 (5 µM, orange line) and (**B**) hGSTP1-1 (0.1mg·ml^{-1}) isoenzyme in the absence (black line) or in the presence of DM62 (5 µM) with mixture GSH + CDNB (2.5 mM + 1 mM respectively, red line) and DM96 (5 µM) with mixture GSH + CDNB (2.5 mM + 1 mM respectively, blue line). Representative spectra from n = 3 independent experiments are presented.

References

[1] D. Matiadis, P.G. Liggri, E. Kritsi, N. Tzioumaki, P. Zoumpoulakis, D.P. Papachristos, G. Balatsos, M. Sagnou, A. Michaelakis, 2021. Curcumin derivatives as potential mosquito larvicidal agents against two mosquito vectors, Culex pipiens and Aedes albopictus, Int. J. Mol. Sci. 22, 8915. https://doi.org/10.3390/ijms22168915.

[2] M.MR. Badal, H.M. Ashekul Islam, M. Maniruzzaman, M. Abu Yousuf, Acidochromic behavior of dibenzylidene cyclohexanone-based bischalcone: experimental and theoretical study, ACS Omega 5 (2020) 22978-22983. https://doi.org/10.1021/acsomega.0c02556.

[3] D. Matiadis, S.T. Ng, E.H.L. Chen, G. Nigianni, V.P. Vidali, A. Canko, R.P.Y. Chen, M. Sagnou, 2021. Synthesis and Biological Evaluation of Hydroxylated Monocarbonyl Curcumin Derivatives as Potential Inducers of Neprilysin Activity, Biomedicines 9, 955. https://doi.org/10.3390/biomedicines9080955.

[4] M. Singh, N. Raghav, Synthesis, docking, and in vitro studies of some substituted bischalcones on acid and alkaline phosphatases, Med. Chem. Res. 23 (2014) 1781-1788. https://doi.org/10.1007/s00044-013-0780-4.

[5] G. Badr, H.I. Gul, C. Yamali, A.A. Mohamed, B.M. Badr, M. Gul, A.A. Markeb, N.A. El-Maali, Curcumin analogue 1, 5-bis (4-hydroxy-3-((4-methylpiperazin-1-yl) methyl) phenyl) penta-1, 4-dien-3-one mediates growth arrest and apoptosis by targeting the PI3K/AKT/mTOR and PKC-theta signaling pathways in human breast carcinoma cells, Bioorg. Chem. 78 (2018) 46-57. https://doi.org/10.1016/j.bioorg.2018.03.006.

[6] M. Homocianu, D. Serbezeanu, A.M. Macsim, T. Vlad-Bubulac, 2020. From cyclohexanone to photosensitive polyesters: Synthetic pathway, basic characterization, and photo-/halochromic properties, J. Mol. Liq. 316, 113888. https://doi.org/10.1016/j.molliq.2020.113888.
